# Characterization of Serum Cytokine Patterns in Frequent-Exacerbation Asthma: Implications for Phenotyping and Management

**DOI:** 10.3390/arm92060047

**Published:** 2024-12-17

**Authors:** Dao Ngoc Bang, Pham Dac The, Pham Thi Kim Nhung, Nguyen Tien Dung, Bach Quoc Tuan, Vu Minh Duong, Le Thi Dieu Hien, Ta Ba Thang

**Affiliations:** 1 Respiratory Center, Military Hospital 103, Vietnam Military Medical University, Hanoi 12100, Vietnam; daongocbang@vmmu.edu.vn (D.N.B.); khanhnhu106@gmail.com (P.T.K.N.); nguyentiendungbv103@vmmu.edu.vn (N.T.D.); tuanqbach@gmail.com (B.Q.T.); 2 Asthma Management Department, Hai Phong International General Hospital, Hai Phong 180000, Vietnam; dr.dacthebmh@yahoo.com; 3 Intensive Care Unit, Military Hospital 103, Vietnam Military Medical University, Hanoi 12100, Vietnam; bsminhduonghscc103@gmail.com; 4 Respiratory Department, Hai Phong University of Medicine and Pharmacy, Hai Phong 180000, Vietnam; doucemd@yahoo.com

**Keywords:** serum cytokine, frequent asthma exacerbations, asthma phenotype

## Abstract

**Highlights:**

**What are the main findings?**

- Asthma patients with frequent exacerbations show significantly elevated serum concentrations of IL-4 and IL-13, along with higher rates of allergic history and specific comorbidities.
- Significant positive correlations exist between inflammatory cytokines (IL-17 and IL-1β) and IgE levels and both IFN-α and TNF-α as well as between FeNO levels.

**What is the implication of the main finding?**

- The distinct cytokine signature in frequent exacerbators suggests potential targets for personalized therapeutic approaches in this high-risk population.
- Integration of cytokine profiling with clinical characteristics may improve patient stratification and guide targeted treatment selection in severe asthma.

**Abstract:**

(1) Background: Asthma exacerbations represent significant clinical events, however, the underlying inflammatory mechanisms and cytokine profiles in patients with frequent exacerbations remain incompletely understood; (2) Methods: In this prospective, cross-sectional study of 120 stable asthma patients, we compared the serum concentrations of eight key cytokines (IL-4, IL-12, IL-13, IL-17, IFN-α, IFN-γ, TNF-α, and IL-1β) between two groups: 60 patients with frequent exacerbations (≥ 2 events per year) and 60 matched controls with few exacerbations (1 event per year); (3) Results: Patients with frequent exacerbations showed significantly higher serum concentrations of IL-4 and IL-13 (*p* < 0.05), along with an increased prevalence of allergic history and comorbidities (chronic rhinosinusitis, GERD, OSA; all *p* < 0.05). The IgE levels correlated positively with IFN-α (rh = 0.26) and TNF-α (rh = 0.29), while the FeNO levels correlated with IL-17 (rh = 0.26) and IL-1β (rh = 0.33) (all *p* < 0.05); (4) Conclusions: Our findings identify a distinct cytokine signature in frequent exacerbators characterized by elevated IL-4 and IL-13 levels. The correlations between specific cytokines and established biomarkers suggest potential mechanisms underlying exacerbation susceptibility, which may inform targeted therapeutic strategies for this high-risk population.

## 1. Introduction

The global burden of asthma continues to rise significantly despite therapeutic advances. Recent epidemiological data indicate an increase in cases from 226.9 million in 1990 to 262.41 million in 2019, with an estimated 461,000 deaths globally in 2019 [1,2]. This trend is particularly concerning in developing nations, where urbanization and environmental changes substantially contribute to disease burden [3,4]. Asthma’s impact extends beyond mortality, significantly affecting healthcare systems and socioeconomic structures worldwide [5].

Despite recent advances in biological agents and targeted therapies, optimal disease control remains challenging. Approximately 17% of asthma patients have difficult-to-treat disease, while 3.7% meet the severe asthma criteria [6,7]. These patients frequently experience exacerbations, leading to accelerated lung function decline and increased healthcare utilization [8,9]. The economic burden is particularly substantial in this population, with frequent exacerbators accounting for a disproportionate share of asthma-related healthcare costs [10,11].

The pathogenesis of frequent exacerbations involves complex inflammatory pathways. Recent evidence highlights the interplay between Th2 and non-Th2 inflammatory responses [12,13], characterized by enhanced airway inflammation and remodeling [14], dysregulated immune responses [15], and variable therapeutic responses [16]. Cytokines serve as crucial mediators in these processes [12,17], with specific patterns potentially defining inflammatory phenotypes [18], predicting exacerbation risk [19], and guiding targeted therapies [16,20].

While numerous studies have investigated cytokine profiles during acute exacerbations [12,18], limited data exist regarding stable asthma patients who experience frequent exacerbations. This study aimed to characterize the serum cytokine concentrations (IL-4, IL-12, IL-13, IL-17, IFN-α, IFN-γ, TNF-α, and IL-1β) in such patients, potentially providing insights into underlying inflammatory mechanisms and therapeutic targets.

## 2. Materials and Methods

### 2.1. Study Design

We conducted a prospective, cross-sectional study at the Asthma Management Department of Hai Phong International General Hospital, Vietnam, between January 2020 and May 2023. The study protocol was designed in accordance with the STROBE guidelines for observational studies (Table S1).

### 2.2. Study Population

The study population consisted of 120 stable asthma patients who experienced their last exacerbation before the time of investigation of at least one month, with the sample size determined using the formula

n = [(Z_1_−α/2)^2^ × σ^2^]/ε^2^μ^2^

where Z_1_−α/2 = 1.96, σ = 17.57% (standard deviation of FVC from Denlinger et al. [8]), μ = 77.45%, and ε = 0.06, yielding a minimum requirement of 55 patients per group. We enrolled 60 patients per group to account for potential data loss, dividing participants into two equal cohorts: Group 1 (n = 60) with frequent exacerbations (≥2 events per year) and Group 2 (n = 60) with few exacerbations (1 event per year) in the most recent year. Eligible participants were required to be ≥16 years old, have a confirmed diagnosis of asthma according to the GINA 2019 guidelines [21], be receiving standardized control treatment, and provide written informed consent. In the study patient group, no patient received biotherapy. Criteria for asthma with few or frequent exacerbations were according to the guidelines of the European Respiratory Society and the American Thoracic Society (2014). We diagnosed asthma exacerbation according to the GINA 2019 guidelines [21]. We excluded patients with acute respiratory infections, coexisting respiratory conditions (e.g., COPD overlap, bronchiectasis), frequent exacerbations related to infections, significant comorbidities as defined by Pavord et al. [20], or those receiving systemic corticosteroid or immunosuppressive therapy.

### 2.3. Clinical Assessment

All participants underwent a comprehensive clinical evaluation following standardized protocols. This assessment encompassed detailed medical and allergic history documentation, thorough family history review, systematic comorbidity screening, and careful documentation of exacerbation frequency over the previous 12 months. Disease control was evaluated using the validated Asthma Control Test (ACT), and treatment intensity was classified according to the GINA step approach [21]. Comorbidities (sinusitis, gastroesophageal reflux disease—GERD, obstructive sleep apnea—OSA, etc.) were diagnosed and stability treated by specialists. We evaluated the allergy history mainly by clinical diagnosis (drug allergy; vaccine hypersensitivity; allergic rhinitis, allergic conjunctivitis, and atopic dermatitis were diagnosed by specialists; urticaria and angioedema related to seafood, insects, etc.), and did not perform allergen testing (prick test, RAST). This standardized assessment protocol ensured consistent data collection across all study participants and aligned with the current clinical practice guidelines.

### 2.4. Laboratory Methods

Comprehensive laboratory analysis encompassed both cytokine quantification and standard biomarker assessment. Serum cytokine levels were measured using a flow cytometry-assisted immunoassay (Bio-Plex system, Bio-Rad, Hercules, CA, USA) with reagents supplied by Bender Medsystems GmbH (Wien, Austria) and Thermo Fisher Scientific. The analysis focused on three distinct cytokine groups: non-Th2-dependent inflammatory cytokines (IL-17, IFN-α, IFN-γ), Th2-dependent inflammatory cytokines (IL-4, IL-13), and inflammatory/regulatory cytokines (IL-12, TNF-α, IL-1β). Additionally, we measured the serum IgE levels, fractional exhaled nitric oxide (FeNO), and conducted standard hematological assessments to provide a comprehensive inflammatory profile for each patient.

### 2.5. Statistical Analysis

Statistical analyses were conducted using SPSS 20.0 and STATA 14.0 software packages, employing a comprehensive analytical approach. Descriptive statistics were used to characterize the demographic and clinical parameters. For continuous variables, we applied either the Student’s *t*-test or Mann–Whitney U test, depending on the data distribution normality, while categorical variables were analyzed using Chi-square tests or Fisher’s exact test when the observation frequencies were less than 5. Associations between variables were assessed using Spearman’s correlation coefficient (rh), with positive values indicating direct correlations and negative values suggesting inverse relationships. Throughout all of the analyses, the statistical significance was defined as *p* < 0.05.

### 2.6. Ethical Considerations

The study protocol was approved by the Ethics Committee of Hai Phong International General Hospital (No. 09/2020/HIH-IRB, 6 January 2020) and conducted in accordance with the Declaration of Helsinki. All participants provided written informed consent prior to study enrollment.

## 3. Results

### 3.1. Subsection

#### 3.1.1. Patient Demographics and Clinical Characteristics

The analysis of 120 asthma patients revealed comparable baseline demographics between the frequent exacerbation (Group 1, n = 60) and few exacerbation groups (Group 2, n = 60), with similar mean ages (50.73 ± 15.05 vs. 50.43 ± 16.56 years, *p* = 0.92), disease duration (26.57 ± 18.32 vs. 29.55 ± 17.62 years, *p* = 0.38), and BMI (22.59 ± 2.86 vs. 22.46 ± 2.45 kg/m², *p* = 0.79) (Table 1). However, Group 1 demonstrated significantly higher rates of personal allergic history (73.3% vs. 36.7%, *p* < 0.001) and key comorbidities including chronic rhinosinusitis (70.0% vs. 45.0%, *p* = 0.006), GERD (38.33% vs. 18.33%, *p* = 0.02), and OSA (33.33% vs. 13.33%, *p* = 0.01). Additionally, Group 1 exhibited poorer disease control with lower mean ACT scores (21.02 ± 3.36 vs. 22.27 ± 3.10, *p* = 0.04) and a higher proportion of patients requiring steps 4–5 treatment (86.67% vs. 76.67%, *p* = 0.16).

#### 3.1.2. Comparison of Serum Cytokine Levels

A comprehensive analysis of serum cytokine profiles revealed distinctive patterns between asthma patients with frequent versus few exacerbations. Notably, the IL-4 levels were significantly elevated in the frequent exacerbation group (median 21.10 pg/mL, IQR 15.99–33.67) compared to those with few exacerbations (median 16.48 pg/mL, IQR 6.75–25.54; *p* = 0.007). Similarly, the IL-13 concentrations were markedly higher in frequent exacerbators (median 9.93 pg/mL, IQR 1.73–13.83 vs. 3.95 pg/mL, IQR 1.73–10.52; *p* = 0.01). While other inflammatory mediators including IL-17 (8.62 vs. 4.86 pg/mL), IL-12 (55.11 vs. 32.24 pg/mL), TNF-α (10.25 vs. 7.30 pg/mL), and IL-1β (1.61 vs. 1.39 pg/mL) showed trends toward elevation in the frequent exacerbation group, these differences did not reach statistical significance (all *p* > 0.05) (Table 2). The levels of IFN-α and IFN-γ remained comparable between groups.

#### 3.1.3. Association Between Cytokines and Allergic History

Within the frequent exacerbation group (n = 60), the analysis of cytokine profiles revealed distinct patterns between patients with (n = 44) and without (n = 16) allergic history. IL-4 demonstrated the most significant difference, with substantially higher levels in allergic patients (22.96 pg/mL, IQR 17.68–35.81) compared to non-allergic patients (18.27 pg/mL, IQR 6.80–23.48; *p* = 0.02) (Table 3). Other Th2-associated cytokines, notably IL-13, showed a trend toward elevation in allergic patients (10.52 pg/mL vs. 4.37 pg/mL, *p* = 0.09), though did not reach statistical significance. Interestingly, while the IL-17 levels were higher in allergic patients (8.78 pg/mL vs. 5.99 pg/mL, *p* = 0.51) and TNF-α showed similar trends (11.59 pg/mL vs. 6.62 pg/mL, *p* = 0.17), these differences did not achieve statistical significance.

#### 3.1.4. Relationship with Disease Severity

The analysis of cytokine profiles in relation to asthma severity within the frequent exacerbation group revealed distinctive patterns between patients with steps 4–5 disease (n = 52) versus steps 2–3 (n = 8). Patients with more severe disease (steps 4–5) showed higher concentrations of several key cytokines, notably IL-4 (22.58 pg/mL, IQR 16.53–33.88 vs. 17.30 pg/mL, IQR 10.05–27.45) and IL-13 (10.39 pg/mL, IQR 1.96–14.38 vs. 2.61 pg/mL, IQR 1.73–12.46). The TNF-α levels were also elevated in the more severe group (10.93 pg/mL vs. 6.62 pg/mL), while IL-17 showed an inverse trend (8.32 pg/mL vs. 10.37 pg/mL) (Table 4). However, it is important to note that none of these differences reached statistical significance (all *p* > 0.05).

#### 3.1.5. Association with Asthma Control

In the frequent exacerbation group, the analysis of cytokine profiles stratified by ACT scores showed that the association of cytokine levels with asthma control assessed by the ACT score were not significant for any of the mediators: IL-17 (9.74 pg/mL, IQR 4.22–17.70 vs. 8.16 pg/mL, IQR 1.47–14.43, *p* = 0.36), IFN-α (0.80 pg/mL, IQR 0.34–1.54 vs. 0.56 pg/mL, IQR 0.40–1.0, *p* = 0.37), TNF-α (11.59 pg/mL vs. 7.75 pg/mL, *p* = 0.14), IL-1β (2.04 pg/mL vs. 1.16 pg/mL, *p* = 0.14), and notably IL-12 (140.96 pg/mL, IQR 29.14–332.72 vs. 45.67 pg/mL, IQR 27.68–257.42, *p* = 0.32). The Th2-associated cytokines showed similar levels between groups IL-4 (21.10 pg/mL vs. 22.58 pg/mL, *p* = 0.93) and IL-13 (9.93 pg/mL vs. 10.06 pg/mL, *p* = 0.59), while the IFN-γ levels remained consistent across both groups (6.54 pg/mL vs. 6.54 pg/mL, *p* = 0.96) (Table 5).

#### 3.1.6. Biomarker Analysis

Analysis of inflammatory biomarkers in the frequent exacerbation group (n = 60) revealed several significant correlations. The serum IgE levels demonstrated weak but significant positive correlations with IFN-α (rh = 0.26, *p* = 0.04) and TNF-α (rh = 0.29, *p* = 0.02). The FeNO concentrations showed significant positive correlations with IL-17 (rh = 0.26, *p* = 0.04) and IL-1β (rh = 0.33, *p* = 0.009), and a trend toward positive correlation with IL-12 (rh = 0.23, *p* = 0.08). Notably, the eosinophil counts showed no significant correlations with any of the measured cytokines: IL-17 (rh = 0.22, *p* = 0.82), IFN-α (rh = 0.006, *p* = 0.96), IFN-γ (rh = 0.17, *p* = 0.18), IL-4 (rh = 0.04, *p* = 0.71), IL-13 (rh = 0.05, *p* = 0.65), IL-1β (rh = 0.03, *p* = 0.80), IL-12 (rh = -0.05, *p* = 0.65), and TNF-α (rh = 0.17, *p* = 0.20) (Table 6).

## 4. Discussion

Our present study provides evidence regarding the inflammatory profiles and clinical characteristics in asthma patients with frequent exacerbations, contributing to our understanding of asthma phenotypes and mechanisms. The demographic patterns and comorbidity profiles observed in this study align with Wang et al.’s [1] systematic review of global asthma burden while offering additional insights specific to frequent exacerbators. The data showed a high prevalence of allergic history in frequent exacerbators (73.3%), consistent with Gerday et al.’s [11] findings on age-specific inflammatory profiles in the atopic–nonatopic asthma paradigm. The observed comorbidity burden including chronic rhinosinusitis (70.0%), GERD (38.33%), and OSA (33.33%) appears consistent with Sun et al.’s [22] observations on comorbidities in asthma control. These findings are further supported by Irina et al.’s [23] observations on respiratory function and align with recent studies by Kaasgaard et al. [24], Salama et al. [25], and Muñoz-Cofré et al. [26] on respiratory impairment and disease monitoring in chronic airway diseases. The data indicate a distinct cytokine signature in frequent exacerbators, characterized by elevated serum concentrations of IL-4 (21.10 pg/mL vs. 16.48 pg/mL, *p* = 0.007) and IL-13 (9.93 pg/mL vs. 3.95 pg/mL, *p* = 0.01). These findings correspond with Sun et al.’s [22] analysis while establishing a potential link between these elevations and exacerbation frequency. The predominance of these Th2-associated cytokines appears consistent with Hizawa’s [12] work on asthma pathogenesis in precision medicine.

Correlation analyses revealed several potentially important relationships in the inflammatory mechanisms of frequent exacerbators. The data showed positive correlations between IgE levels and both IFN-α (rh = 0.26, *p* = 0.04) and TNF-α (rh = 0.29, *p* = 0.02), suggesting possible interactions between allergic and inflammatory pathways, as described in McIntyre and Viswanathan’s [15] work on asthma phenotype complexity. The correlations between FeNO levels and inflammatory cytokines, particularly with IL-17 (rh = 0.26, *p* = 0.04) and IL-1β (rh = 0.33, *p* = 0.009), may indicate the involvement of non-Th2 inflammatory pathways, consistent with Salter et al.’s [18] findings. The lack of significant correlations between eosinophil counts and cytokine levels, despite elevated Th2 cytokines, suggests that stable-state eosinophil counts may not necessarily reflect the underlying inflammatory activity, supporting Hynes and Pavord’s [20] observations on inflammatory mechanisms in severe asthma. The stratified analysis by disease severity and control status, while not achieving statistical significance, indicates potential relationships between disease severity and inflammatory complexity, aligning with De Ferrari et al.’s [8] work on disease burden mechanisms and Ishmael et al.’s [9] findings on molecular pathways. These observations may have implications for the management of frequent exacerbators, as the cytokine patterns and biomarker correlations could inform therapeutic approaches, as discussed in the recent literature [13,14,16,17,19]. The data suggest that multiple inflammatory pathways may be involved, potentially supporting the consideration of comprehensive assessment and combination therapeutic approaches, as described by Talbot et al. [10] in their review of severe asthma management.

## 5. Conclusions

Our study demonstrated several key findings regarding cytokine profiles in stable asthma patients with frequent exacerbations. Most notably, the IL-4 and IL-13 concentrations were significantly elevated in frequent exacerbators and showed specific associations with allergic phenotypes. Furthermore, we identified significant correlations between inflammatory markers, with the IgE levels correlating positively with both IFN-α and TNF-α, and the FeNO levels showing positive associations with IL-17 and IL-1β. These findings suggest a distinct inflammatory signature in frequent exacerbators that could inform targeted therapeutic approaches [9,13,20].

## 6. Limitations

The single-center design and sample size, which was just sufficient according to the power calculations, may limit the generalizability of our findings. The study’s cross-sectional nature prevented the evaluation of temporal changes in cytokine patterns and treatment responses. Technical constraints included one-time cytokine measurements and the inability to assess changes following treatment modifications. Additionally, our focus on stable patients excluded insights from acute exacerbations, and the regional nature of our population may affect the broader applicability of our findings.

## 7. Future Directions

Several key areas warrant further investigation based on our findings. Longitudinal studies with larger, multi-center cohorts are needed to validate our observations and evaluate cytokine pattern changes over time, particularly during and between exacerbations. Future research should focus on integrating additional biomarkers for comprehensive inflammatory profiling and developing predictive models to guide personalized treatment approaches. Investigating targeted therapies based on specific cytokine profiles and assessing preventive strategies for frequent exacerbators could advance precision medicine approaches in severe asthma [6,7,19]. These directions align with recent developments in precision medicine and could significantly improve our understanding and management of exacerbation-prone phenotypes.

## Figures and Tables

**Table 1:** Characteristics of the study population.

Characteristics		Group 1 (n_1_ = 60)	Group 2 (n_2_ = 60)	*p*
Age ($\bar{X}\;$± SD) (years)		50.73 ± 15.05	50.43 ± 16.56	0.92
Gender:	-Male: n (%)	17 (28.33)	26 (43.33)	0.09
	-Female: n (%)	43 (71.67)	34 (56.67)	
Allergic history	Personal; n (%)	44 (73.3)	22 (36.7)	<0.001 *
	Family; n (%)	14 (23.3)	15 (25.0)	0.83
Asthma onset (years old)	≤12; n (%)	34 (56.67)	40 (66.67)	0.26
	>12; n (%)	26 (43.33)	20 (33.33)	
Smoking: n (%)		10 (16.7)	18 (30.0)	0.084
Step of asthma	Step (2–3); n (%)	8 (13.33)	14 (23.33)	0.16
	Step (4–5); n (%)	52 (86.67)	46 (76.67)	
Duration of asthma (years) ($\bar{X}\;$± SD)		26.57 ± 18.32	29.55 ± 17.62	0.38
Comorbidities:	-Sinusitis: n (%)	42 (70.0)	27 (45.0)	0.006 *
	-GERD: n (%)	23 (38.33)	11 (18.33)	0.02 *
	-OSA: n (%)	20 (33.33)	8 (13.33)	0.01 *
	-Diabetes: n (%)	6 (10.0)	6 (10.0)	1
	-Hypertension: n (%)	9 (15.0)	6 (10.0)	0.41
ACT score	<20	26 (43.33)	17 (28.33)	0.16
	20–24	16 (26.67)	16 (26.67)	
	≥25	18 (30.0)	27 (45.0)	
	($\bar{X}\;$± SD)	21.02 ± 3.36	22.27 ± 3.10	0.04 *
BMI ($\bar{X}\;$± SD) (kg/m^2^)		22.59 ± 2.86	22.46 ± 2.45	0.79

Baseline demographic and clinical characteristics of asthma patients with frequent exacerbations (Group 1, n = 60) compared to those with few exacerbations (Group 2, n = 60). Data are presented as mean ± SD or number (percentage). * *p* < 0.05 indicates statistical significance. BMI: body mass index, GERD: gastroesophageal reflux disease, OSA: obstructive sleep apnea, ACT: asthma control test.

**Table 2:** Serum cytokine levels of the study groups.

Cytokine Level Median (p_25_–p_75_) (pg/mL)	Group 1 (n_1_ = 60)	Group 2 (n_2_ = 60)	*p*
IL-17	8.62 (2.88–14.92)	4.86 (1.47–13.95)	0.11
INF-α	0.57 (0.40–1.43)	0.50 (0.40–1.43)	0.67
INF-γ	6.54 (5.05–11.95)	5.57 (5.05–9.96)	0.63
IL-4	21.10 (15.99–33.67)	16.48 (6.75–25.54)	0.007 *
IL-13	9.93 (1.73–13.83)	3.95 (1.73–10.52)	0.01 *
IL-12	55.11 (28.17–269.03)	32.24 (17.22–271.04)	0.06
TNF-α	10.25 (4.76–18.92)	7.30 (3.53–17.88)	0.18
IL-1β	1.61 (0.81–2.68)	1.39 (0.81–3.76)	0.78

Comparison of the serum cytokine concentrations between asthma patients with frequent exacerbations (Group 1, n = 60) and those with few exacerbations (Group 2, n = 60). Values are presented as median with interquartile range (25th–75th percentiles). * *p* < 0.05 indicates statistical significance.

**Table 3:** Relation between the serum cytokine concentrations and allergic history.

Cytokine Level Median (p_25_–p_75_) (pg/mL)	Asthma Patients with Frequent Exacerbations (n = 60)		
	No Allergy (n = 16)	Allergy (n = 44)	*p*
IL-17	5.99 (2.13–14.24)	8.78 (3.55–15.21)	0.51
IFN-α	0.40 (0.29–0.89)	0.59 (0.40–1.47)	0.11
IFN-γ	5.81 (4.83–8.52)	7.51 (5.05–16.02)	0.11
IL-4	18.27 (6.80–23.48)	22.96 (17.68–35.81)	0.02 *
IL-13	4.37 (1.73–10.52)	10.52 (3.48–14.94)	0.09
IL-12	174.96 (32.47–265.83)	45.09 (27.44–269.03)	0.48
TNF-α	6.62 (3.53–12.61)	11.59 (5.26–19.05)	0.17
IL-1β	1.61 (0.69–2.16)	1.63 (0.81–2.68)	0.41

Comparison of the serum cytokine levels between asthma patients with frequent exacerbations with (n = 44) and without (n = 16) allergic history. Values are presented as median with interquartile range (25th–75th percentiles). * *p* < 0.05 indicates statistical significance.

**Table 4:** Relationship between the serum cytokine concentrations and asthma steps.

Cytokine Level Median (p_25_–p_75_) (pg/mL)	Asthma Patients with Frequent Exacerbations (n = 60)		
	Asthma Steps (2, 3) (n = 8)	Asthma Steps (4, 5) (n = 52)	*p*
IL-17	10.37 (6.42–14.92)	8.32 (2.13–15.12)	0.63
IFN-α	0.68 (0.4–0.98)	0.57 (0.4–1.47)	0.88
IFN-γ	5.57 (4.83–15.58)	7.02 (5.05–11.95)	0.63
IL-4	17.30 (10.05–27.45)	22.58 (16.53–33.88)	0.20
IL-13	2.61 (1.73–12.46)	10.39 (1.96–14.38)	0.17
IL-1β	1.61 (0.99–2.16)	1.61 (0.81–2.68)	0.68
IL-12	242.21 (33.52–333.52)	249.53 (28.17–262.23)	0.37
TNF-α	6.62 (4.40–14.39)	10.93 (4.76–19.05)	0.29

Serum cytokine concentrations in asthma patients with frequent exacerbations according to the asthma severity steps: steps 2–3 (n = 8) versus steps 4–5 (n = 52). Values are presented as the median with interquartile range (25th–75th percentiles).

**Table 5:** Relationship between the serum cytokine concentrations and ACT score.

Cytokine Level Median (p_25_–p_75_) (pg/mL)	Asthma Patients with Frequent Exacerbations (n = 60)		
	ACT < 20 (n = 26)	ACT ≥ 20 (n = 34)	*p*
IL-17	9.74 (4.22 -17.70)	8.16 (1.47–14.43)	0.36
IFN-α	0.80 (0.34–1.54)	0.56 (0.40–1.0)	0.37
IFN-γ	6.54 (5.05–11.95)	6.54 (5.05–14.82)	0.96
IL-4	21.10 (15.44–33.52)	22.58 (16.53–33.81)	0.93
IL-13	9.93 (1.73–10.87)	10.06 (2.44–14.39)	0.59
IL-12	140.96 (29.14–332.72)	45.67 (27.68–257.42)	0.32
TNF-α	11.59 (7.98–19.72)	7.75 (3.53–18.78)	0.14
IL-1β	2.04 (0.81–2.68)	1.16 (0.81–2.68)	0.14

Serum cytokine levels in asthma patients with frequent exacerbations stratified by ACT (Asthma Control Test) scores: ACT < 20 (n = 26) versus ACT ≥ 20 (n = 34). Values are presented as the median with interquartile range (25th–75th percentiles).

**Table 6:** Correlation between the serum cytokine concentrations and eosinophil count, IgE, FeNO.

Indices			IL-17	IFN-α	IFN-γ	IL-4	IL-13	IL-1β	IL-12	TNF-α
Asthma patients with frequent exacerbations (n = 60)	Eosinophil	r_h_	0.22	0.006	0.17	0.04	0.05	0.03	−0.05	0.17
		*p*	0.82	0.96	0.18	0.71	0.65	0.80	0.65	0.20
	IgE	r_h_	0.14	0.26	0.17	0.15	0.21	0.14	−0.054	0.29
		*p*	0.28	0.04	0.19	0.25	0.10	0.27	0.68	0.02 *
	FeNO	r_h_	0.26	0.06	0.14	0.004	0.07	0.33	0.23	0.05 *
		*p*	0.04	0.64	0.30	0.97	0.59	0.009	0.08	0.72

Correlations between the serum cytokine concentrations and eosinophil count, IgE levels, and FeNO in asthma patients with frequent exacerbations (n = 60). rh represents the Spearman’s correlation coefficient. * *p* < 0.05 indicates statistical significance. FeNO: fractional exhaled nitric oxide.

## Data Availability

The data supporting this research are available from the authors on reasonable request.

## Supplementary Materials

The following supporting information can be downloaded at: https://www.mdpi.com/article/10.3390/arm92060047/s1, Table S1: STROBE Statement.
