# Supplementary material for: Characterization of Serum Cytokine Patterns in Frequent-Exacerbation Asthma: Implications for Phenotyping and Management
Source: Adv Respir Med. 2024 Dec 17;92(6):538–47. doi: 10.3390/arm92060047 (PMC11673841; doi:10.3390/arm92060047)
Supplement: Supplementary file 1 [file arm-92-00047-s001.zip › arm-3324576-supplementary.pdf]

STROBE Statement—checklist of items that should be included in reports of observational studies

	Item No.	Recommendation	Page No.	Relevant text from manuscript
**Title and abstract**	1	(*a*) Indicate the study's design with a commonly used term in the title or the abstract	1	Characterization of Serum Cytokine Patterns in Fre-quent-Exacerbation Asthma: Implications for Phenotyping and Management
		(*b*) Provide in the abstract an informative and balanced summary of what was done and what was found	1	Abstract section with background, methods, results, conclusions
**Introduction**				
Background/rationale	2	Explain the scientific background and rationale for the investigation being reported	1-2	Introduction paragraphs 1-4: Global burden of asthma, challenges in management, role of cytokines
Objectives	3	State specific objectives, including any prespecified hypotheses	2	Introduction final paragraph: "This study aims to characterize serum cytokine concentrations..."
**Methods**				
Study design	4	Present key elements of study design early in the paper	2	Section 2.1: "prospective, cross-sectional study"
Setting	5	Describe the setting, locations, and relevant dates, including periods of recruitment, exposure, follow-up, and data collection	2	Section 2.1: "Hai Phong International General Hospital, Vietnam, between January 2020 and May 2023"
Participants	6	*Cross-sectional study*—Give the eligibility criteria, and the sources and methods of selection of participants	2	Section 2.2: Detailed inclusion/exclusion criteria, sample size calculation
Variables	7	Clearly define all outcomes, exposures, predictors, potential confounders, and effect modifiers. Give diagnostic criteria, if applicable	2-3	Sections 2.3-2.4: Clinical assessment and laboratory methods
Data sources/ measurement	8	For each variable of interest, give sources of data and details of methods of assessment (measurement). Describe comparability of assessment methods if there is more than one group	3	Section 2.4: "flow cytometry-assisted immunoassay (Bio-Plex system)"
Bias	9	Describe any efforts to address potential sources of bias	2	Section 2.2: Exclusion criteria to minimize confounding
Study size	10	Explain how the study size was arrived at	2	Section 2.2: Formula provided with parameters

Continued on next page

Quantitative variables	11	Explain how quantitative variables were handled in the analyses. If applicable, describe which groupings were chosen and why	3	Section 2.5: Statistical analysis methods
Statistical methods	12	*Cross-sectional study*—If applicable, describe analytical methods taking account of sampling strategy	3	Section 2.5: SPSS 20.0 and STATA 14.0, specific tests described
**Results**				
Participants	13	Report numbers of individuals at each stage of study—eg numbers potentially eligible, examined for eligibility, confirmed eligible, included in the study, completing follow-up, and analysed	3	Section 3.1.1: 120 total participants (60 per group)
Descriptive data	14	Give characteristics of study participants (eg demographic, clinical, social) and information on exposures and potential confounders	5-6	Table 1: Demographics and clinical characteristics
Outcome data	15	*Cross-sectional study*—Report numbers of outcome events or summary measures	7-10	Tables 2-6: Cytokine levels and correlations
Main results	16	Give unadjusted estimates and, if applicable, confounder-adjusted estimates and their precision (eg, 95% confidence interval). Make clear which confounders were adjusted for and why they were included	3-5	Sections 3.1.2-3.1.5: Key findings with statistical significance
Other analyses	17	Report other analyses done—eg analyses of subgroups and interactions, and sensitivity analyses	4-5	Section 3.6: Biomarker correlations
**Discussion**				
Key results	18	Summarise key results with reference to study objectives	10-11	Discussion opening paragraphs
Limitations	19	Discuss limitations of the study, taking into account sources of potential bias or imprecision. Discuss both direction and magnitude of any potential bias	12	Section 5.2: Study limitations
Interpretation	20	Give a cautious overall interpretation of results considering objectives, limitations, multiplicity of analyses, results from similar studies, and other relevant evidence	11	Discussion main body: Context with existing literature
Generalisability	21	Discuss the generalisability (external validity) of the study results	12	Section 5.2: Discussion of limitations affecting generalizability
**Other information**				
Funding	22	Give the source of funding and the role of the funders for the present study and, if applicable, for the original study on which the present article is based	12	Funding statement: No financial support

*Give information separately for cases and controls in case-control studies and, if applicable, for exposed and unexposed groups in cohort and cross-sectional studies.

Note: An Explanation and Elaboration article discusses each checklist item and gives methodological background and published examples of transparent reporting. The STROBE checklist is best used in conjunction with this article (freely available on the Web sites of PLoS Medicine at http://www.plosmedicine.org/, Annals of Internal Medicine at http://www.annals.org/, and Epidemiology at http://www.epidem.com/). Information on the STROBE Initiative is available at www.strobe-statement.org.
